# Decrypting the H-NS-dependent regulatory cascade of acid stress resistance in *Escherichia coli*

**DOI:** 10.1186/1471-2180-10-273

**Published:** 2010-10-29

**Authors:** Evelyne Krin, Antoine Danchin, Olga Soutourina

**Affiliations:** 1 Unité de Plasticité du Génome Bactérien, Institut Pasteur, France; 2 AMAbiotics 5, rue Henri Desbruères, 91030 Evry Cedex, France; 3 Unité des Bactéries Anaérobies et Toxines, Institut Pasteur, France; 4 Université Paris 7-Denis Diderot, 75205 Paris, France

## Abstract

**Background:**

H-NS regulates the acid stress resistance. The present study aimed to characterize the H-NS-dependent cascade governing the acid stress resistance pathways and to define the interplay between the different regulators.

**Results:**

We combined mutational, phenotypic and gene expression analyses, to unravel the regulatory hierarchy in acid resistance involving H-NS, RcsB-P/GadE complex, HdfR, CadC, AdiY regulators, and DNA-binding assays to separate direct effects from indirect ones. RcsB-P/GadE regulatory complex, the general direct regulator of glutamate-, arginine- and lysine-dependent acid resistance pathways plays a central role in the regulatory cascade. However, H-NS also directly controls specific regulators of these pathways (e.g. *cadC*) and genes involved in general stress resistance (*hdeAB, hdeD, dps, adiY*). Finally, we found that in addition to H-NS and RcsB, a third regulator, HdfR, inversely controls glutamate-dependent acid resistance pathway and motility.

**Conclusions:**

H-NS lies near the top of the hierarchy orchestrating acid response centred on RcsB-P/GadE regulatory complex, the general direct regulator of glutamate-, arginine- and lysine-dependent acid resistance pathways.

## Background

In *Escherichia coli*, complex cellular responses are controlled by networks of transcriptional factors that regulate the expression of a diverse set of target genes, at various hierarchical levels. H-NS, a nucleoid-associated protein, is a top level regulator affecting the expression of at least 250 genes, mainly related to the bacterial response to environmental changes [1]. Among its various targets, it regulates in opposite directions the flagella-dependent motility and the acid stress resistance [1]; the first via the control of *flhDC *master flagellar operon by acting both directly and indirectly via regulators HdfR and RcsB [2-6]; the second by repressing the genes involved in three amino acid decarboxylase systems, dependent on glutamate, lysine and arginine, via the RcsB-P/GadE regulatory complex [6]. In this regulatory process H-NS represses the expression of *gadE *(encoding the central activator of the glutamate-dependent acid resistance pathway) both in a direct and an indirect way, via EvgA, YdeO, GadX and GadW [1,7,8], while it decreases *rcsD *expression, essential to the phosphorylation of RcsB (the capsular synthesis regulator component) required for the formation of the regulatory complex with GadE [6]. In the glutamate pathway, the RcsB-P/GadE regulatory complex controls the expression of two glutamate decarboxylase paralogues GadA and GadB, the glutamate/gamma-aminobutyrate antiporter GadC, two glutamate synthase subunits GltB and GltD, the acid stress chaperones HdeA and HdeB, the membrane protein HdeD, the transcriptional regulator YhiF (DctR) and the outer membrane protein Slp [6]. The complex also induces an arginine decarboxylase, AdiA, and an arginine:agmatine antiporter, AdiC (YjdE), essential for arginine-dependent acid resistance. Finally, the complex regulates a lysine decarboxylase, CadA, and a cadaverine/lysine antiporter, CadB, essential for lysine-dependent acid resistance [1,6,9]. Apart from the *gadBC *operon, the most important genes involved in acid resistance are present within the acid fitness island (AFI), a 15 kb region both repressed by H-NS and under the control of RpoS [10,11]. Recent global chromatin immunoprecipitation studies revealed that H-NS binds to several loci within this region, including *hdeABD *[12,13]. However, neither AdiY, the main regulator of the arginine-dependent response that controls *adiA *and *adiC *expression [14,15] nor CadC, the main regulator of lysine-dependent response controlling *cadBA *[16], were yet found among the identified H-NS targets.

In the present study, we aimed at further characterizing the H-NS-dependent cascade governing acid stress resistance pathways to identify the missing intermediary regulator(s) or functional protein(s) controlled by H-NS and to define the interplay between the different regulators and their targets.

## Methods

### Bacterial strains and plasmids

Bacterial strains and plasmids used in this study are listed in Table 1. Mutants were constructed by replacing the entire gene of interest with an antibiotic cassette using the CF10230 strain, as previously described [17]. These mutations, as well as their Miki and Keio collection counterparts from NBRP (NIG, Japan): *E. coli *[18,19] were subsequently transduced into FB8 *hns*::*Sm *derivative strains, using P1*vir *phage. When required, antibiotics were added: ampicillin (100 μg ml^-1^), streptomycin (10 μg ml^-1^), kanamycin (40 μg ml^-1^), tetracycline (15 μg ml^-1^).

**Table 1:** Bacterial strains and plasmids used in this study

Strain or plasmid	Genotype or description	Reference or source
**Strains**		
JD21162	KP7600 (*F- lacIQ lacZdeltaM15 galK2 galT22 lambda- in (rrnD-rrnE*)1, W3110 derivative) *ydeP *::*Km*	[19]
JD24946	KP7600 (*F- lacIQ lacZdeltaM15 galK2 galT22 lambda- in (rrnD-rrnE*)1, W3110 derivative) *yhiM *::*Km*	[19]
JD25275	KP7600 (*F- lacIQ lacZdeltaM15 galK2 galT22 lambda- in (rrnD-rrnE*)1, W3110 derivative) *hdeA *::*Km*	[19]
JD26576	KP7600 (*F- lacIQ lacZdeltaM15 galK2 galT22 lambda- in (rrnD-rrnE*)1, W3110 derivative) *ydeO*::*Km*	[19]
JD27509	KP7600 (*F- lacIQ lacZdeltaM15 galK2 galT22 lambda- in (rrnD-rrnE*)1, W3110 derivative) *dps *::*Km*	[19]
JW5594	BW25113 *(rrnB ΔlacZ4787 HsdR514 Δ(araBAD)567 Δ(rhaBAD)568 rph-1*) ΔaslB ::*Km*	[18]
JW2366	BW25113 *(rrnB ΔlacZ4787 HsdR514 Δ(araBAD)567 Δ(rhaBAD)568 rph-1*) ΔevgA ::*Km*	[18]
EP247	W3110 *cadC1*::Tn10	[41]
FB8	Wild type	[42]
BE1411	FB8 *hns*::*Sm*	[43]
BE2823	FB8 *hns*::*Sm ΔrcsB *::*Km*	[6]
BE2825	FB8 *hns*::*Sm ΔhdfR *::*Tet*	This study
BE2826	FB8 *hns*::*Sm dps *::*Km*	FB8 *hns::Sm *× P1 JD27509
BE2827	FB8 *hns*::*Sm rpoS 359 *::*Km*	This study
BE2828	FB8 *hns*::*Sm yhiM *::*Km*	FB8 *hns::Sm *× P1 JD24946
BE2829	FB8 *hns*::*Sm ΔevgA *::*Km*	FB8 *hns::Sm *× P1 JW2366
BE2830	FB8 *hns*::*Sm ΔaslB *::*Km*	FB8 *hns::Sm *× P1 JW3772
BE2831	FB8 *hns*::*Sm ydeP *::*Km*	FB8 *hns::Sm *× P1 JD21162
BE2832	FB8 *hns*::*Sm ydeO *::*Km*	FB8 *hns::Sm *× P1 JD26576
BE2836	FB8 *hns*::*Sm ΔhdeA *::*Km*	FB8 *hns::Sm *× P1 JD25275
BE2837	FB8 *hns*::*Sm ΔadiY *::*Tet*	This study
BE2939	FB8 *hns*::*Sm cadC1*::Tn10	FB8 *hns::Sm *× P1 EP247
**Plasmids**		
pDIA640	pet22b ::*hdfR *with C terminal His tag	This study
pDIA642	pet16b ::*rcsB_D56E _*with N terminal His tag	[6]
pDIA645	pet22b ::*gadE *with C terminal His tag	[6]
pDIA646	pet16b ::*adiY *with N terminal strep tag	This study

### Resistance to low pH

The experiment was performed at least twice, as previously described [6].

### RNA preparation and Real-time quantitative RT-PCR

The experiment was performed twice, as previously described [6]. Primers used in real-time quantitative RT-PCR experiments are listed in Additional file 1.

### Protein purification

H-NS-His6 was purified as previously described [20]. Recombinant proteins HdfR-His6, His6-RcsB_D56E_, GadE-His6 and Strep-AdiY were purified as previously described [6].

### Gel mobility shift assays

Gel shift assays were performed with 0.1 ng [γ^32^P]-labelled probe DNA with purified HdfR-His6, His6-RcsB_D56E _(mimicking phosphorylated and activated RcsB), GadE-His6 and Strep-AdiY proteins as previously described [6,10]. For competitive gel mobility shift assays with purified H-NS protein 100-200 ng PCR fragments of target promoter regions and 270-200 ng of competitor DNA fragments, obtained by digestion of pBR322 plasmid with *Taq*I and *Ssp*I restriction enzymes, were incubated for 15 min at room temperature with H-NS in the previously described reaction mixture [21]. Protein-DNA complexes were resolved on 3% or 4% MetaPhor agarose gel. Primers used in gel mobility shift assays are listed in Additional file 2.

## Results

### Determination of new H-NS targets involved in the regulation of glutamate-dependent acid resistance

As H-NS strongly represses the glutamate-dependent acid stress response, there is a very low level of survival after acid stress in the FB8 wild-type context [6]. As a consequence, H-NS targets involved in this process are only expressed when *hns *is removed. To find further H-NS-dependent intermediary actors of glutamate-dependent acid resistance, several of the H-NS induced targets, identified in a previous transcriptome analysis [1] and related either to acid stress resistance or to information pathways, were deleted in an *hns*-deficient strain. We looked for a decreased glutamate-dependent acid resistance, in comparison to that displayed in the parent *hns*-deficient strain. Different extent of decrease in resistance to acidic conditions was observed with deletion of several genes known to be related to acid stress response including *dps *(coding for the Dps protein - DNA-binding protein of starved cells), *rpoS *(coding for the RNA polymerase sigma-38 factor), *yhiM *(coding for an inner membrane protein), *evgA *(coding for a transcriptional activator), *ydeP *(coding for a putative anaerobic dehydrogenase) and *ydeO *(coding for a transcriptional regulator, which is a target of sRNA OxyS) (Table 2), suggesting a role in the H-NS-controlled glutamate-dependent acid resistance. Furthermore, a reduced resistance was also observed with genes, not previously associated with acid stress, such as *aslB *(coding for an anaerobic sulfatase-maturating enzyme homolog) and *hdfR *(coding for the H-NS-dependent *flhDC *regulator) (Table 2). However, the single deletion of several genes including *evgA*, *ydeP*, *ydeO *and *aslB *in *hns *background only slightly affected the acid stress survival, suggesting their redundant function in this H-NS-dependent process.

**Table 2:** Glutamate-dependent acid resistance of *E. coli *strains

Strain (relevant genotype)	Glutamate-dependent acid resistance (% survival)
FB8 (wild-type)	0.1
BE1411 (*hns*::*Sm*)	51.7
BE2823 (*hns*::*Sm ΔrcsB*)	< 0.001

BE2825 (*hns*::*Sm ΔhdfR*)	12.5
BE2826 (*hns*::*Sm dps*::*Km*)	20.1
BE2827 (*hns*::*Sm rpoS*)	27.5
BE2828 (*hns*::*Sm yhiM*::*Km*)	24.2
BE2829 (*hns*::*Sm ΔevgA*)	32.0
BE2831 (*hns*::*Sm ydeP*::*Km*)	35.6
BE2832 (*hns*::*Sm ydeO*::*Km*)	38.2
BE2830 (*hns*::*Sm ΔaslB*)	38.6
BE2837 (*hns*::*Sm ΔadiY*)	5.4
BE2939 (*hns*::*Sm cadC1*::Tn10)	58.1

Data are the mean values of two independent experiments that differed by less than 20%.

### Determination of H-NS targets involved in arginine and/or lysine-dependent acid resistance

We wondered whether the new genes identified as H-NS-controlled, but unrelated to regulation by the RcsB-P/GadE complex, also play a role in the arginine and lysine-dependent acid resistance pathways. We performed acid stress assays in the presence of these amino acids with *hns*-deficient strains also deleted in these genes. Only the deletion of *dps *led to dramatically low survival rate in the presence of arginine and lysine, while the deletion of *hdeA *resulted in a 5-fold decreased survival rate in the presence of arginine and slightly modified survival rate in the presence of lysine (Table 3). Although the arginine and lysine-dependent acid resistance pathways are regulated by H-NS [1], it is not known whether AdiY and CadC, the specific regulators of these pathways respectively, are controlled by H-NS. Real-time quantitative RT-PCR experiments were carried out on *adiY *and *cadC *with RNA isolated from FB8 wild-type and *hns*-deficient strains. We observed that the *adiY *and *cadC *RNA level increased five-fold in the *hns *mutant (Table 4), suggesting that they may mediate the effect of H-NS on arginine and lysine-dependent acid stress resistance. To further investigate the role of *adiY *and *cadC *in the H-NS-dependent control of acid resistance, each gene was deleted in an *hns *background and the acid resistance assays were performed in the presence of arginine, glutamate and lysine. In the absence of *adiY*, much fewer bacteria survived in the presence of glutamate and arginine, but not in the presence of lysine, while the *cadC *deletion led to extreme acid stress sensitivity only in the presence of lysine (Table 2 and 3). This suggests a role of CadC regulator in the H-NS regulation of the lysine-dependent acid stress resistance and a role of AdiY regulator in the arginine- and glutamate-dependent pathways.

**Table 3:** Arginine and lysine-dependent acid resistance of *E. coli *strains

Strain (relevant genotype)	Arginine-dependent acid resistance (% survival)	Lysine-dependent acid resistance (% survival)
FB8 (wild-type)	0.23	0.05
BE1411 (*hns*::*Sm*)	24.50	7.64
BE2823 (*hns*::*Sm ΔrcsB*)	4.44	1.00
BE2826 (*hns*::*Sm Δdps*)	0.11	0.28
BE2836 (*hns*::*Sm ΔhdeA*)	5.11	5.37
BE2837 (*hns*::*Sm ΔadiY*)	1.80	7.30
BE2939 (*hns*::*Sm cadC1*::Tn10)	24.24	0.001

Percentage survival is calculated as 100 × number of c.f.u. per ml remaining after 2 hours low pH treatment in the presence of arginine or lysine, divided by the initial c.f.u. per ml at time zero. Data are the mean values of two independent experiments that differed by less than 15%.

**Table 4:** Quantitative RT-PCR analysis on H-NS targets involved in acid stress resistance

	Expression ratio				
	
Gene	*hns/wild-type*	*hns gadE*/wild-type	*hns rcsB*/wild-type	*hns hdfR*/wild-type	*hns adiY*/wild-type
Glutamate-dependent specific pathway					
***gadA^1^***	137.21	nd	Nd	150.93	41.31
***dctR^1^***	34.66	nd	Nd	34.32	8.84
***yhiM***	10.75	3.41	3.40	10.90	11.36
***aslB***	12.92	0.66	1.10	0.69	1.32
***gltD^1^***	1.68	nd	Nd	0.48	0.52

Arginine-dependent specific pathway					
***adiA***	16.89	nd	Nd	nd	0.70
***adiC***	11.62	nd	Nd	nd	1.41

Lysine-dependent specific pathway					
***cadC***	4.62	5.77	6.38	nd	nd

General acid stress resistance pathway					
***hdeA^1^***	32.37	nd	Nd	41.20	6.55
***hdeD***	18.96	nd	Nd	17.57	5.89
***adiY***	5.08	5.00	5.00	nd	nd

nd: non-determined.

1: Since several genes are organized in operon and/or are highly homologous to each other, results obtained with *gadA *also corresponds to *gadBC*; with *gltD *to *gltB*; with *hdeA *to *hdeB*; with *dctR *to *slp*.

Quantitative RT-PCR were performed on total RNA isolated from exponential growth phase cultures. Standard deviations were less than 20% of the mean.

### Identification of the target genes for major regulators

To decipher the regulatory hierarchy in acid stress resistance involving several new H-NS controlled regulators, the mRNA level of target genes was compared between wild-type and *hns*, *hns rcsB*, *hns gadE*, *hns hdfR, hns adiY *mutant strains, using real-time quantitative RT-PCR (Table 4). In particular, we compared the expression ratio between a double mutant and the wild-type strain with that for *hns*-deficient and the wild-type strain. H-NS having negative effect on target genes, these genes are strongly derepressed in *hns *mutant in comparison with wild-type strain. If this strong H-NS repressive effect is abolished in the absence of a regulator negatively controlled by H-NS, we can conclude that this deleted regulator has positive effect on target gene expression and may be an intermediary actor in H-NS-dependent control for this target, as previously shown [6].

It was found that RcsB and GadE upregulate, at the similar level, newly identified genes involved in acid stress resistance pathways dependent on glutamate (*yhiM *and *aslB*), but these two regulators did not affect the expression of regulatory genes, *cadC *and *adiY *(Table 4). Neither RcsB nor GadE controlled *hdfR *regulatory gene expression (data not shown), suggesting that the *hdfR *is not the target of RcsB-P/GadE complex. We found that HdfR controlled only the expression of *aslB *and *gltBD *in the glutamate-dependent acid stress resistance regulon (Table 4). As expected, AdiY strongly affected *adiA *and *adiC *expression, and also the expression of some genes related to the glutamate specific pathway (*aslB*, *gadA, gadBC, gltBD*, and *slp-dctR*) and to general acid resistance (*hdeAB *and *hdeD*) (Table 4). These results demonstrated a multiple control of several target genes involving different regulators acting independently from each other.

### Identification of the new targets directly controlled by RcsB-P/GadE complex

**Figure 1:**
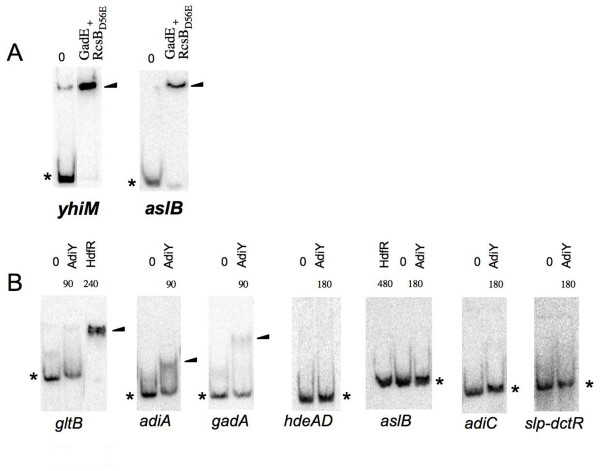
**Gel mobility shift assays with GadE/RcsB_D56E _complex, HdfR and AdiY**. A. Gel mobility shift assays with GadE/RcsB_D56E _complex and new DNA targets. Proteins were incubated with DNA targets during 30 min at 25°C in the final reaction mixture volume of 15 μl. 900 ng of each GadE and RcsB_D56E _protein are used for *yhiM *and *aslB*. B. Gel mobility shift assays with HdfR or AdiY proteins. Quantities of purified HdfR or AdiY proteins are indicated above each lane (in ng). Gel mobility shift assays (A and B) were performed with 0.1 ng [γ^32^P]-labelled DNA fragment and loaded on a 6% polyacrylamide native gel. An arrow points out the position of the DNA-regulatory protein complex. An asterisk marks the position of the unbound probe.

Gel mobility shift assays were performed with a mixture of purified RcsB_D56E _and GadE proteins to know whether the regulatory complex directly controlled *yhiM *and *aslB*. It was established that the RcsB_D56E_/GadE regulatory complex binds to the promoter regions of the two genes (Figure 1A), demonstrating the direct control by the RcsB-P/GadE complex.

### Identification of the targets directly controlled by HdfR or AdiY

Real-time quantitative RT-PCR analysis showed that HdfR regulates *aslB *and *gltBD*, while AdiY regulates several genes involved in acid stress resistance (*adiA*, *adiC*, *aslB*, *gadA, gadBC, gltBD, hdeAB, hdeD *and *slp-dctR*) (Table 4). To establish whether these regulators control the expression of these genes by direct binding to their promoter regions, gel mobility shift assays were performed with purified HdfR and AdiY proteins. It was found that HdfR binds to the promoter region of *gltBD *and that AdiY binds to the promoter regions of *gltBD, adiA *and *gadABC *(Figure 1B). However, no band shift was observed even with higher concentration of regulator with HdfR on the promoter region of *aslB *and with AdiY on the promoter regions of *adiC*, *aslB, hdeABD *and *slp-dctR *(Figure 1B), suggesting an indirect regulation for these genes.

### Identification of the targets directly controlled by H-NS

**Figure 2:**
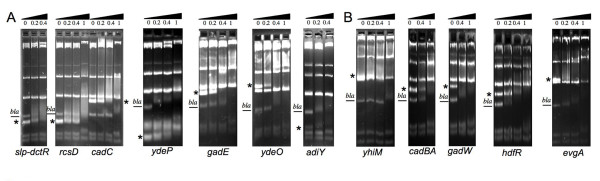
**Competitive gel mobility shift assay with H-NS, target promoter fragments and restriction fragments derived from plasmid pBR322**. The cleaved plasmid and promoter fragments were incubated with the indicated concentrations of purified H-NS protein (in μM). After protein-DNA complex formation, the fragments were resolved on a 3% (A) or 4% (B) MetaPhor agarose gel. An asterisk indicates the position of the target promoter fragments. "*bla*" indicates the *bla *promoter (positive control), the other fragments of plasmid DNA correspond to negative controls. The specific binding of H-NS is observed when bands corresponding to *bla *and target promoter disappear with increasing concentration of H-NS, the H-NS-DNA complex being difficult to visualize under these conditions.

H-NS modulates the expression of several regulators controlling acid stress resistance including HdfR, RcsD, EvgA, YdeO, YdeP, GadE, GadW, GadX, AdiY and CadC. However, the direct control by H-NS has not yet been established for the majority of these regulators, except for GadX [22] and HdfR [3]. Furthermore, *slp-dctR *and *yhiM *could also be directly repressed by H-NS, as deletion of their regulators, RcsB-P/GadE complex and/or AdiY, in *hns*-deficient strain was not sufficient to restore their wild-type mRNA level (Table 4) [6]. Competitive gel mobility shift assays were performed with purified H-NS protein on PCR fragments, corresponding to assayed promoters, and restriction fragments derived from the pBR322 plasmid, used as negative competitors for binding to H-NS protein except for one 217-bp DNA fragment corresponding to the *bla *promoter used as positive internal control [21]. A preferential binding of H-NS was observed to the promoter regions of *adiY, cadBA, cadC, evgA, gadE*, *gadW, hdfR, rcsD, slp-dctR, ydeO, ydeP, yhiM*, confirming the direct control by H-NS of these genes (Figure 2).

## Discussion

**Figure 3:**
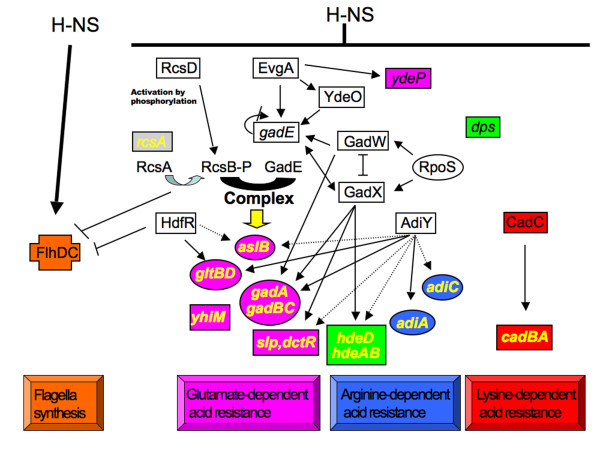
**Model of the H-NS-dependent regulatory network in flagella and acid stress control**. At the top, H-NS positively controls motility and represses acid stress resistance. Genes in cross symbol are directly activated by H-NS; in rectangle: directly repressed by H-NS; in circle: indirectly repressed by H-NS. Regulatory proteins are indicated with upper case. Orange filling: flagellum synthesis process; Pink filling: glutamate-dependent acid resistance process; Blue filling: arginine-dependent acid resistance process; Red filling: lysine-dependent acid resistance process; Green filling: genes involved in three different acid resistance processes. Gene names in yellow indicate the direct targets of RcsB-P/GadE complex placed at the centre of this regulatory cascade. A positive effect on transcription is indicated by arrows and a negative regulatory effect is indicated by blunt ended lines. Direct regulation is indicated by solid lines. Indirect regulation is indicated by dashed lines. Previously published results are included in the scheme: [1-3,5-7,10,16,32-40].

H-NS regulates directly and indirectly the RcsB-P/GadE complex, that is located at the centre of the acid resistance network as well as control of motility (Figure 3). Furthermore, H-NS modulates the level of several regulatory proteins, unrelated to this complex (e.g. CadC, AdiY, HdfR) (Table 4 and Figure 2) [3]. Among them, only HdfR was previously known as a H-NS target [3]. The present study revealed that, in addition to its role in motility control, HdfR regulates the glutamate-dependent acid resistance pathway, directly inducing *gltBD *and indirectly controlling *aslB *(Table 4 and Figure 1, 3). All the results presented in this work were integrated together with previously published data, to propose a model of the complex H-NS-dependent regulatory network governing motility and acid stress resistance processes in *E. coli *(Figure 3). The new characterized H-NS targets, CadC and AdiY, have no effect on motility (data not shown) and are involved in the H-NS-dependent regulation of lysine and arginine-dependent response to acid stress, respectively (Table 3). Furthermore, we found that AdiY is also involved in glutamate-dependent response to acid stress (Table 2). It directly or indirectly regulates several genes specific to this response including *aslB, gltBD, gadA, gadBC, slp-dctR *or having more global role in acid stress resistance such as *hdeAB *and *hdeD *(Table 4). Interestingly, we demonstrated that H-NS has a direct control effect on the *cadBA *promoter (Figure 2), in accordance with the previous suggestion of a competition between the CadC activator and H-NS for binding to this promoter region [23]. In addition to its role in the repression of major regulators at high levels of the hierarchy, we have shown that H-NS is able to directly affect acid stress circuits repressing the transcription of several structural genes (e.g. *yhiM, slp, dctR*) (Figure 2). This is in agreement with the proposed competition between activation by specific regulators and repression by H-NS, in several bacterial systems [24,25]. The results of present study point out the essential role for several intermediary players within H-NS-dependent regulatory network and suggest an accessory role for other regulators in acid stress response. Indeed, the EvgA-YdeO regulatory pathway plays a secondary modulator role in the glutamate-dependent acid stress response, in comparison to H-NS. In the same means, AslB and YdeP, two anaerobic enzymes, may have a redundant function in this stress response.

Among the H-NS-regulated genes, we showed that the acid stress chaperones HdeA and HdeB that solubilized periplasmic protein aggregates at acid pH [26] are involved in all three pathways of acid stress response. However, their impact is low in the arginine- and lysine-dependent pathways (Table 3), while they are essential in the glutamate-dependent pathway [27]. This could be explained by the fact that arginine and lysine amino acids are able to strongly oppose protein aggregation [28]. By contrast, we found that the expression of the *dps *gene, directly regulated by H-NS and known to protect cells against multiple stresses [29], is essential to lysine- and arginine-dependent responses to acid stress, while its role is less important during the glutamate-dependent response (Table 2 and 3). This implies that the induced glutamate-dependent response provides sufficient cell protection, restricting Dps to a marginal role. This is consistent with the observation that glutamate is widely distributed amino acid representing approximately 15--45% in the dietary protein content and plays a key physiological role in gastrointestinal tract [30].

Within this frame of thought, the glutamate decarboxylase system would be the most efficient acid resistance mechanism [31]. This could also explain why three regulators H-NS, HdfR and RcsB are directly involved in the control of both glutamate-dependent acid stress response and the flagellum biosynthesis. Indeed, as flagellum is a high consumer of ATP and leads to proton entrance during its motor functioning, it is necessary to stop this process to limit cytoplasmic acidification in bacteria and to redirect energy to mechanisms of resistance to stress. Furthermore, the flagellar filaments bear strong antigenic properties in contact with host. We can suppose that these complex high-organized regulations allow a faster adaptation to environmental variations and an increase in survival under adverse conditions found in gastric environment contributing to the virulence of gastrointestinal bacteria, examplified by enteroinvasive *E. coli *HN280 [32].

## Conclusion

In *E. coli*, the control of acid stress resistance is achieved by the concerted efforts of multiple regulators and overlapping systems, most of the genes directly involved in acid resistance being both controlled by RcsB-P/GadE complex and by at least one other regulator such as H-NS, HdfR, CadC or AdiY.

## Authors' contributions

EK conceived the study, performed all experiments and drafted the manuscript. AD helped to finalize the manuscript and to place it in perspective, OS helped to analyse the data and to draft the manuscript. All authors read and approved the final manuscript.

## Supplementary Material

Additional File 1 **List of primers used in real-time quantitative RT-PCR experiments**. Click here for file

Additional File 2 **List of primers used for gels retardation assay**. Click here for file
