# Supplementary material for: Decrypting the H-NS-dependent regulatory cascade of acid stress resistance in Escherichia coli
Source: BMC Microbiol. 2010 Oct 29;10:273. doi: 10.1186/1471-2180-10-273 (PMC2984483; doi:10.1186/1471-2180-10-273)
Supplement: Additional File 1 — List of primers used in real-time quantitative RT-PCR experiments. [file 1471-2180-10-273-S1.DOC]

**Additional file 1.** List of primers used in real-time quantitative RT-PCR experiments.

16Srt3: gtaagggccatgatgacttg

16srt5: tcagctcgtgttgtgaaatg

Adiart3: tcagcatcaaaccttgttcg

Adiart5: cggtagtcgtcggtacttcc

Adicrt3: ACCAGAACCAGCCAAATACG

Adicrt5: TCGTCCTGCTGAACATTGTC

Adiyrt3: AGTGCGTTCTCTGTGCTGAC

Adiyrt5: AGTCCTGCGGCTACAACAGT

Aslbrt3: CGTTGTGCTCAACAATCACC

Aslbrt5: GCGATATTCGACCACTGGAT

Cadcrt3: GCGTCAACTTACCCTTGAGC

Cadcrt5: CTCTGCGTCACAACGTGATT

Dpsrt3: tcagccagttctttcaggtg

Dpsrt5: gtgtagctctggggaccact

Gadabrt3: tcgggtccataaacaactga

Gadabrt5: aggcaaaccaacggataaac

Gltdrt3: cgggatgtagttgtgtaccg

Gltdrt5: ctgaagatccgcaaaattga

Hdeart3: gccaggaaatcttcacaggt

Hdeart5: ttggtggtctgcttcttctg

Hdedrt3: caatgacgatatccagcaca

Hdedrt5: catttattgccggtttgttc

Hdfrt3: GCCGTGAAAGTGTTGTGCTA

Hdfrt5: GCAACAGATTGCGATGCTTA

Dctrrt3: tgcagctcagaacctgatgt

Dctrrt5: tgttccgcggactatcacta

Yhimrt3: tgccagtagccagacttttg

Yhimrt5: tagtcagttccgatgcaagc
