# Supplementary material for: Decrypting the H-NS-dependent regulatory cascade of acid stress resistance in Escherichia coli
Source: BMC Microbiol. 2010 Oct 29;10:273. doi: 10.1186/1471-2180-10-273 (PMC2984483; doi:10.1186/1471-2180-10-273)
Supplement: Additional File 2 — List of primers used for gels retardation assay. [file 1471-2180-10-273-S2.DOC]

**Additional file 2**. List of primers used for gels retardation assay

| **Primers** | **Sequence** | **Reference** | **Size of fragment** |
| --- | --- | --- | --- |
| gadX-1 | 5'-CGCAATAATATATTGGCTGT-3' | [1] | 325bp |
| gadX-2 | 5'-ATGTAGTGATTGCATAGTTG-3' | [1] |
| gadE-1 | 5'-GTCGAAACAAGGAGACTCGA-3' | [1] | 285bp |
| gadE-2 | 5'-TGTTCAATATAGTAAACGCC-3' | [1] |
| EvgAshift5 | 5'-atttaaatgaggtgcaaaaaaaa-3' | [2] | 310bp |
| EvgAshift3 | 5’-ttgcgttcatagattattcc-3’ | [2] |
| YdePshift5 | 5’-ttatatatccatgttggcga-3’ | [2] | 113bp |
| YdePshift3 | 5’-GATTCAATTTTTTTCTTCAT-3’ | [2] |
| YdeOshift5 | 5'-tactgattaacgatttttaa-3' | [2] | 340bp |
| YdeOshift3 | 5'-ACAGAACAAACGAGCGACAT-3' | [2] |
| GadWshift5 | 5'-taagctatacgctgtgcgaa-3' | [2] | 276bp |
| GadWshift3 | 5'-TCGTTATGCAGCGATATTTT-3' | This study |
| HdfRshift5 | 5'-atcctgcacgctcctaattc-3' | [2] | 261bp |
| HdfRshift3 | 5'-AAGGTTCACACCCAGTTGAT-3' | This study |
| RcsDshift5 | 5'-cgcataatttccagcaatct-3' | [2] | 190bp |
| RcsDshift3 | 5'-agggcagtataaagggtaca-3' | [2] |
| Slptdctrshift5 | 5'-gaataaaaatagattttatg-3' | [2] | 196bp |
| Slpdctrshift3 | 5'-ttaaaaccataatagatatt-3' | [2] |
| AdiYshift5 | 5'-tggccttaggccttttgagt-3' | [2] | 168bp |
| AdiYshift3 | 5'-gttttactaatagtacaaat-3' | [2] |
| YhiMshift5 | 5'-aatgatacttgtctgaaaaa-3' | [2] | 300bp |
| YhiMshift3 | 5'-taatgataatttcatcctgt-3' | [2] |
| AslBshift5 | 5'-ttttgttttctgtgcatttc-3' | [2] | 120bp |
| AslBshift3 | 5'-ggtcgctccttgcctgaagt-3' | [2] |
| gltB-1 | 5'-CAAAATTACCGAAATTTCAT-3' | [1] | 435bp |
| gltB-2 | 5'-TTCGCATCGGTTAATACGGT-3' | [1] |
| hde-1 | 5'-CGACACTGAGGTTATAACCTGG-3' | [1] | 162bp |
| hde-2 | 5'-ATGCCAAAAACGCGTCTAAG-3' | [1] |
| CadCshift5 | 5'-ttgctcatgcaaagactaac-3' | [2] | 285bp |
| CadCshift3 | 5'-cattcgaaaagggaatgatg-3' | [2] |
| AdiAshift5 | 5'-gcgccgccagacgtttcccg-3' | [2] | 282bp |
| AdiAshift3 | 5'-GCGTTACCGACCCAGGTGTC-3' | [2] |
| AdiCshift5 | 5'-tttttaaccttaacgaagag-3' | [2] | 156bp |
| AdiCshift3 | 5'-GCATCAGCATCCGAAGACAT-3' | [2] |
| CadBAshift5 | 5’-taatttttattacataaatttaac-3’ | [2] | 259bp |
| CadBAshift3 | 5’-taatttcatttttgaatttggagtc-3’ | [2] |
| gadA-203 | 5’-GAACTCCTTAAATTTATTTG-3’ | [1] | 185bp |
| gadA-201 | 5’-TTTGGGCGATTTTTATTACG-3’ | [3] |

1. Hommais F, Krin E, Coppee JY, Lacroix C, Yeramian E, Danchin A, Bertin P: **GadE (YhiE): a novel activator involved in the response to acid environment in *Escherichia coli***. *Microbiology* 2004, **150**(1):61-72.

2. Krin E, Danchin A, Soutourina O: **RcsB plays a central role in H-NS-dependent regulation of motility and acid stress resistance in *Escherichia coli***. *Res Microbiol* 2010, **161**(5):363-371.

3. Castanie-Cornet MP, Foster JW: **Escherichia coli acid resistance: cAMP receptor protein and a 20 bp cis-acting sequence control pH and stationary phase expression of the gadA and gadBC glutamate decarboxylase genes**. *Microbiology* 2001, **147**(Pt 3):709-715.
